# 

**Published:** 1895-03-30

**Authors:** 


					(J (AaA Vt(1 7 Maeoh 30, 1SB5,
r-jf^ WW Wl? EXTRA NURSING supplement.
IRL*\ x* Va? I MMntM 1 ft a CI A nAat fnAA
?? 1, MM
Far Annum, 10a, 6d., post free.
Monthly Farts, 6d.; post free, 8d.
Ditto per Annum, 8s. 6d? postlfree.
ffllY/CH HQS Pi TAL
No. 444. Vol. XVII. WITH EXTRA NURSING SUPPLEMENT. Saturday, March 30th, 1895.

				

